# Intrinsic direct air capture

**DOI:** 10.1039/d5sc06099k

**Published:** 2025-09-10

**Authors:** Austin McDannald, Daniel W. Siderius, Brian DeCost, Kamal Choudhary, Diana L. Ortiz-Montalvo

**Affiliations:** a Materials Measurement Science Division, National Institute of Standards and Technology Gaithersburg MD USA austin.mcdannald@nist.gov; b Chemical Sciences Division, National Institute of Standards and Technology Gaithersburg MD USA; c Materials Science and Engineering Division, National Institute of Standards and Technology Gaithersburg MD USA

## Abstract

How can you tell if a sorbent material will be good for any gas separation process – without having to do detailed simulations of the full process? We present new metrics to evaluate solid sorbent materials for Direct Air Capture (DAC), a particularly challenging gas separation problem, based entirely on intrinsic properties of the sorbent material. These new metrics provide a theoretical upper bound on CO_2_ captured per energy as well as a theoretical upper limit on the purity of the captured CO_2_. These metrics apply to any adsorption-refresh cycle design. The only inputs are the path adsorption-refresh cycle in terms of thermodynamic variables and the intrinsic materials properties (primarily the equilibrium uptake, and heat capacity) along that path. In this work we demonstrate the use of these metrics with the example of temperature–pressure swing refresh cycles. To apply these metrics on a set of examples, we first generated approximations of the necessary materials properties for 11 660 metal–organic framework materials (MOFs). We find that the performance of the sorbents is highly dependent on the path through thermodynamic parameter space. These metrics allow for: (1) finding the optimum materials given a particular refresh cycle, and (2) finding the optimum refresh cycles given a particular sorbent. Applying these metrics to the database of MOFs lead to the following insights: (1) start cold – the equilibrium uptake of CO_2_ diverges from that of N_2_ at lower temperatures, and (2) selectivity of CO_2_*vs.* other gases at any one point in the cycle does not matter – what matters is the relative change in uptake along the cycle.

## Abstract 2

How can you tell if a sorbent material will be good for any gas separation process – without detailed simulations of the full process? We present new metrics to evaluate solid sorbent materials based on intrinsic properties of the sorbent material.

## Graphical abstract

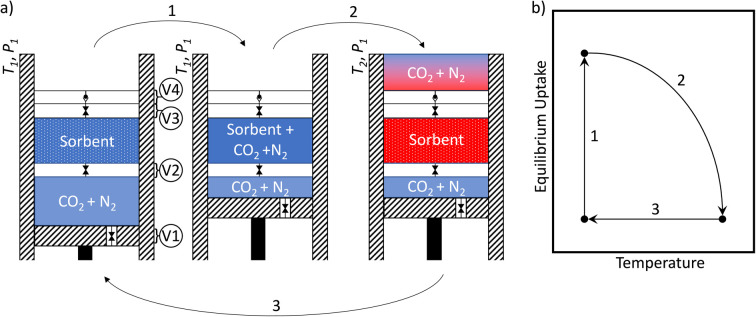

## 1 Introduction

Direct Air Capture (DAC) seeks to capture CO_2_ from the atmosphere. As such it is one of the most challenging industrial gas separation problems, since the target gas species (CO_2_) has a concentration of 410 μmol mol^−1^ (ppm).^1^ There are pilot DAC facilities, such as ClimeWorks, Carbon Engineering, and Global Thermostat, that have recently started operations.^2^ The scale of proposed fully realized DAC implementations imposes strict and challenging demands on the performance of these facilities, especially in terms of energy.^3^ For these facilities, the performance metrics and design considerations of DAC are well defined. These include:

• The CO_2_ capture efficiency: how much CO_2_ is captured per energy used.

• The CO_2_ capture output: how much CO_2_ is captured in a given length of time.

• The refresh cycle time: the time it takes to perform one refresh cycle.

• The purity of the captured CO_2_: the concentration of the CO_2_ in the output.

• The sorbent stability: the structural stability – especially (for DAC applications) in the presence of water vapor.

• The sorbent synthesizability: the ease and economic viability of synthesizing the sorbent on an industrial scale.

• The sorbent longevity: the time or number of refresh cycles the sorbent can experience before degrading to the point of needing to be replaced.

However, many of these performance metrics are difficult to calculate or predict without detailed simulations of a particular process or the construction of a pilot plant. It can often be unclear what sorbent material properties will lead to good facility performance. In the synthesis of solid sorbents, much emphasis was put on achieving the synthesis of materials with high gravimetric or volumetric surface areas.^4,5^ The motivation for this seems to be that higher surface areas should allow for more interactions, since the sorbent interacts with the sorbate (gas to be adsorbed) at the surface. But these purely geometric measures incorporate no information about the interaction between the sorbent and sorbate. Another popular characteristic for comparing sorbents is working capacity. This is the difference in equilibrium uptake gas between two conditions – the amount of gas adsorbed and then desorbed on one refresh cycle. Working capacity does provide some information about the interaction between the sorbent and the sorbate, but working capacity is not enough to determine if a material will perform well as a sorbent. A sorbent with a small working capacity – but with little energy needed to cycle, or that could be cycled quickly – could be much better in a DAC facility than one with a large working capacity. Furthermore, working capacity is typically presented for conditions with a single sorbate (*e.g.* a pure CO_2_ environment at different partial pressures). Air, however, is a mixed gas. Therefore, the mixed gas adsorption behavior must be considered in DAC applications.

There have been efforts to develop performance indicators for sorbents. Jain *et al.*^6^ developed a set of heuristics based on parameters such as particle size and time in the adsorption bed to aid in the design of pressure swing adsorption systems. Neumann *et al.*^7^ use a detailed model of the adsorption process in a gas separation column to calculate performance metrics for the sorbent. Similarly, Young *et al.*^8^ developed a machine learning model as a surrogate for detailed simulations of adsorption columns which allows them to screen many materials for that process, and provide insights into the sorbent and process design. While these can provide an accurate estimate of the performance metrics for that process, it depends on particular design choices of the adsorption process – such as the feed gas velocity, adsorption column design, and packing density. Ajenifuja *et al.*,^9^ created a model for screening sorbents for temperature swing based adsorption. While this model is more directly relevant than using the selectivity or working capacity, it assumes that the adsorption system is a packed powder bed and, therefore, depends on extrinsic parameters such as the packing density. It further assumes that the refresh cycle is purely a temperature swing, which limits the generalizability to other thermodynamic spaces and can therefore not account for different paths through those spaces during refresh cycles, such as the increasingly popular temperature-vacuum swing adsorption cycles,^10^ or to electro-swing adsorption cycles.^11^ Recently, Charalambous *et al.*^12^ developed a holistic platform for evaluating carbon capture systems. They do this by not only considering some of the aforementioned traditional sorbent performance indicators, but also additionally considering performance indicators of the process, a techno-economic assessment, and a life-cycle assessment. At the materials level, they primarily use the ratio of Henry's constants to indicate the performance, then feed the materials properties information into detailed simulations of an adsorption column process. The performance metrics we present in this work could readily be included into the platform developed by Charalambous *et al.*^12^ at the materials level to provide more informative sorbent performance indicators, and aid in the design of the processes – without the need to perform detailed process simulations. In general, the previous methods evaluating the performance of adsorption systems depend on extrinsic factors (such as the design of the gas separation column and consequential fluid dynamics). Furthermore, these previous methods typically only apply to a particular choice of refresh cycle – they would be difficult to generalize to other choices of refresh cycle, or even to other choices of process parameters.

In this work, we develop sorbent performance metrics based on intrinsic material properties. These metrics directly generalize to any gas adsorption process when described in terms of thermodynamic parameters. Specifically, we develop the theoretical upper limit on the amount of CO_2_ captured per unit energy (which we term the Capture Efficiency) and the purity of the captured CO_2_. For illustrative purposes, we derive this model using the example of temperature vacuum swing adsorption cycle, which we visualize with an idealized piston. While this model is not how DAC facilities are likely to operate in practice, this framing of the problem elucidates all the relevant thermodynamic terms. Much in the same way the Carnot cycle is a theoretical upper limit on the efficiencies of heat engines, these metrics are the theoretical upper limit of the capture efficiency and purity for gas separation processes based on adsorption. Inverting the capture efficiency is equivalent to estimating the theoretical lower limit of the energy cost needed to capture an amount of CO_2_.

For the purposes of this work, we will approximate air as 400 μmol mol^−1^ of CO_2_ with the balance of N_2_. This binary mixture is the minimum complexity example that still demonstrates the full considerations for this analysis technique, and was chosen as the target sorbate of interest, CO_2_, and the majority component of air, N_2_. However, we also show how this analysis can be extended to consider arbitrary gas mixtures. Practical DAC systems would need to consider the effects of H_2_O in the inlet stream. Such practical considerations could be included in more detailed studies of individual sorbents if there were pair potentials for H_2_O of sufficient accuracy, or otherwise accurate sources of mixed gas adsorption behavior for mixtures that include H_2_O. For the present high-throughput study, the next section details both how we generate the necessary materials data for this analysis considering our binary mixture of CO_2_ and N_2_, as well as how we develop the thermodynamic analysis of an intrinsic DAC cycle.

## 2 Modeling

### 2.1 Generating the data

The goal of the metrics we develop in these works is provide a fair comparison between sorbent materials, and between thermodynamic refresh cycles. As such the thermodynamic analysis we use is based on intrinsic materials properties. Chief among those is the equilibrium uptake of each of the gas species in the mixture. In addition, we also require the heat of adsorption for each gas species to compute the heating and cooling duties. Lastly, because many refresh cycles include a temperature swing, we also require the heat capacities (*C*_V_) of sorbent materials in order to determine the energy requirements associated with temperature changes during the CO_2_ capture cycle. As a set of example materials, we generate all the needed materials property data for the 11 660 metal–organic framework materials (MOFs) that are in both the Cambridge Structural Database (CSD) database^13^ and the Computation Ready Experimental MOF (CoRE MOF) database^14^ of experimentally realized MOFs. It is worth noting that while we apply these metrics to MOFs, they could just as easily be applied to zeolites, covalent-organic framework materials, hybrid systems, or any other solid sorbent system.

#### 2.1.1 Equilibrium adsorption properties

The equilibrium uptake of CO_2_ in a sorbent is a function not only of temperature (*T*) and the partial pressure of CO_2_ (*P*_CO_2__), but also the partial pressure (*i.e.*, composition) of the other gases – in this case the partial pressure of N_2_ (*P*_N_2__). That is *n*_CO_2__(*T*, *P*_CO_2__, *P*_N_2__), and similarly *n*_N_2__(*T*, *P*_CO_2__, *P*_N_2__).

However, there is very little multicomponent gas adsorption data available,^15^ and even single-component adsorption measurements for most sorbents exhibit poor reproducibility.^16^ We therefore turn to simulations to estimate the equilibrium uptake. However, to fully characterize the temperature, pressure, and composition dependence of equilibrium uptake, we would require simulations across the relevant ranges of those variables for every material (11 600 in total) in our study, incurring significant computational expense. Therefore, we exploit a series of simplifications that reduce the necessary computational effort. As a first simplification, we approximate the multicomponent equilibrium uptake using Ideal Adsorbed Solution Theory (IAST)^17^ applied to the simulation-derived, single-component adsorption isotherms. This simplification eliminates the need for multicomponent simulations across the necessary range of gas compositions, allowing us to focus on single-component simulations at appropriate pressure.

Our second simplification is based on the relevant pressure range of our cycle analysis. Since the typical pressures for DAC are low (near or sub-atmospheric), we can approximate the isotherms for many sorbents by the (linear) Henry's Law Isotherm, where *K*_H_ is the Henry's law constant. As is well-known,^18^ the *K*_H_ for a particular sorbate, sorbent, and temperature combination may be computed using the rapid (compared to full Monte Carlo simulation) Widom-insertion approach,^19^ which we implemented in the Free Energy and Advanced Sampling Simulation Toolkit (FEASST).^20,21^

To complete the data set for equilibrium uptake, we must also address the temperature-dependence of *K*_H_. Recently, Siderius *et al.*^22^ demonstrated that *K*_H_ may be represented by a high-order polynomial extrapolation function of inverse temperature (
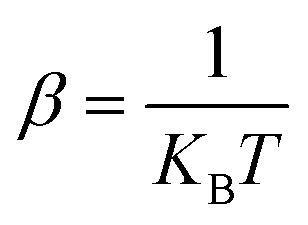
, where *K*_B_ is the Boltzmann constant), where the extrapolation coefficients are related to moments of the internal energy observed in a Widom-insertion calculation. Thus, as a third simplification we additionally used FEASST to collect the extrapolation coefficients for *K*_H_, ultimately yielding *K*_H_ for relevant temperatures by a simple polynomial function of *β*. We note that this technique, *via* an appropriate derivative of *K*_H_, also provides the heat of adsorption at infinite dilution^22^ (*q*^∞^_ads_). Thus, for each sorbent considered, we ran two Widom-insertion simulations, one for CO_2_ at 350 K and the other for N_2_ at 300 K, yielding *K*_H,i_ and *q*^∞^_ads,i_ (where i = CO_2_ or N_2_) as polynomial functions of *β*. The CO_2_ simulation was done at higher temperature since that sorbate is likely to reach saturation at higher temperatures. Details of these calculations and example scripts are given in Section 2 of the SI.

Given the simplifications noted above, we ran additional simulations to identify thermodynamic boundaries within which those simplifications are adequate or appropriate. Our first consideration is the linearity simplification, particularly for N_2_ uptake. As an approximation to atmospheric conditions, we will consider an input total pressure of 101.3 kPa (1 atm) with a CO_2_ concentration of 400 μmol mol^−1^ (400 ppm) with the balance of N_2_. Under this approximation of atmospheric conditions the partial pressure of CO_2_ is very low and thus likely well within the linear regime of the isotherm, since most of the total pressure is due to the balance of N_2_. We also expect that the pure N_2_ isotherms for most sorbents are also linear up to 101.3 kPa, since the temperatures examined herein are far above the critical temperature of N_2_. To verify that the N_2_ isotherm is sufficiently linear, we performed Grand Canonical Monte Carlo (GCMC) simulations of pure N_2_ in each sorbent material at *P* = 101.3 kPa for comparison with the linear isotherm at 300 K. The linear isotherm based on *K*_H,N_2__ was considered an adequate approximation when its uptake ±10% was within the 95% confidence interval of the uptake obtained by direct GCMC calculation. Those materials where these two predictions do not agree will require more sophisticated approximations, and should be the subject of future studies. Second, since CO_2_ will reach saturation at higher temperatures than N_2_, we also considered the adsorption saturation of CO_2_ to ensure that the linear isotherm does not grossly overestimate the CO_2_ uptake. To estimate the adsorption saturation of CO_2_ in each material, we ran an additional simulation of pure CO_2_ in each sorbent at 350 K and at increasing pressure until the uptake of CO_2_ converges. Then, we restrict the DAC cycle analysis to temperatures where *K*_H_ × *P*_CO_2__ is below the saturation uptake, *n*_sat,CO_2__; this effectively sets a lower bound on the valid temperature range. Detailed description of these simulations are provided in Section 2 of the SI. Third, the extrapolation functions that represent *K*_H_ must be restricted to thermodynamically consistent temperatures; that is, *K*_H_ must be a monotonic, increasing function of temperature. This condition may impose an upper limit on the temperature range of the extrapolated *K*_H_ and, hence, the CO_2_ and/or N_2_ linear isotherms.

Ultimately, the molecular simulations listed above yield the pure-component Henry Law isotherms of CO_2_ and N_2_ for each material at arbitrary temperature, subject to restrictions on the temperature range that follow from thermodynamic and saturation-loading considerations. With these isotherms in hand, we can then use IAST to obtain the mixed gas equilibrium uptake at arbitrary temperatures and partial pressures of each gas and, with linear *K*_H_ isotherms, IAST can be applied analytically. In the SI we derive the equilibrium uptake along the desorption path in Step 2 of the intrinsic refresh cycle. We show this derivation for three cases: analytically solving IAST for the binary mixture of CO_2_ and N_2_, a correction for finite volume of the desorption chamber, and the generic case of non-linear adsorption behavior in multi-component gas mixtures (given the known functions *n*_A_(*T*, *P*_A_, *P*_B_, *P*_C_, …), *n*_B_(*T*, *P*_A_, *P*_B_, *P*_C_, …), *n*_C_(*T*, *P*_A_, *P*_B_, *P*_C_, …), where A, B, C, …refer to arbitrary gas species).

#### 2.1.2 Sorbent heat capacity

In ref. 23, Moosavi *et al.* trained machine learning models, specifically XGBoost^24^ models, to predict the *C*_V_ of MOFs and zeolites. These models were trained against a computational workflow that involved density functional theory calculations and molecular dynamics simulations for the force of atom displacements, phonon modes of the material, and subsequent calculations of the *C*_V_s. The computational workflow allows for the integration of the phonon modes across temperature to obtain the temperature dependence of the *C*_V_s. In their work, Moosavi *et al.* used separate ensembles of XGBoost models to predict the *C*_V_s at several different temperatures (250 K to 400 K in 25 K increments). In this work, we use the pre-trained ensembles of 100 XGBoost models for each temperature from ref. 23 and their code repository^25^ for the predictions and uncertainties of the *C*_V_s.

To interpolate and extrapolate the *C*_V_ predictions to arbitrary temperatures, we use a heteroscedastic Gaussian Process regressor (hGPR), implemented using ref. 26. This hGPR propagates the uncertainties from the *C*_V_ predictions from the XGBoost model to make new predictions of the *C*_V_ with quantifiable uncertainty at arbitrary temperatures. This hGPR model therefore allows us to predict the *C*_V_ of the MOF sorbents at each step of the refresh path discussed in the next section.

### 2.2 Thermodynamic model

#### 2.2.1 Model

**Fig. 1:**
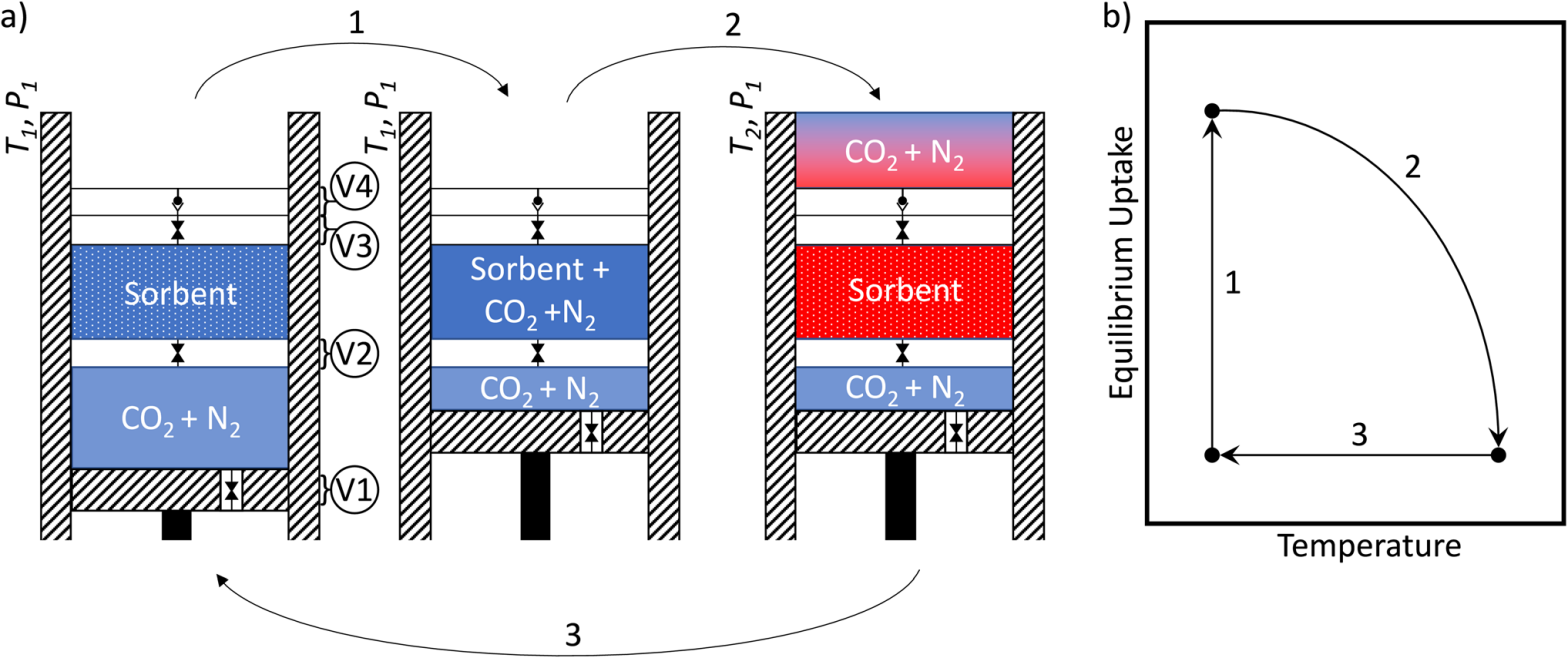
(a) A diagram of a piston model for adsorption process. This diagram shows an example temperature swing adsorption cycle. The diagonal hashed rectangles represent perfectly insulative walls. The valves are labeled V1 to V4. The steps 1 to 3 are steps of the example temperature swing adsorption cycle, starting at temperature *T*_1_, ending at temperature *T*_2_, at pressure *P*_1_. The blue-to-red color scheme represents the low-to-high temperature. (b) A sketch of the example temperature swing refresh path in temperature and equilibrium uptake space. The steps 1 to 3 correspond to those in part (a).

In order to envision all the thermodynamic terms, it is helpful to consider a model. For this, we will use an idealized piston in Fig. 1.

This idealized piston system allows for the visualization of all of the terms in the energy and mass balances. Real DAC systems are typically packed columns of sorbent, and the performance of these systems depends on many extrinsic factors such as the column diameter, the flow rate, and the packing density in the column. By considering this idealized piston system, we avoid those factors and instead depend on intrinsic material properties. This idealized piston makes three strong assumptions: (1) the system reaches thermodynamic equilibrium at each step in the process, (2) the system does not consider any extrinsic factors like sorbent particle packing fraction, (3) as the gas is desorbed it leaves the system through a zero-volume check-valve as discussed in more detail below (see Section 1.2 of the SI for correction terms for this). With those assumptions, this system will find the theoretical upper limit on the capture efficiency as well as the purity of the captured gas. The utility of this work then is twofold. The intrinsic refresh analysis can first be used to find optimal materials for a given thermodynamic process. Secondly, this intrinsic refresh analysis can be used to find optimal thermodynamic processes for a material. In the derivations in the next section we show how this work could be extended to consider real-world extrinsic design considerations such as packing fraction and waste heat recovery.

The surfaces of the piston chamber are assumed to be perfectly insulative; the piston is assumed to be mass-less, and the valves and check-valves are assumed to have no volume. The refresh cycle proceeds as follows:

Step 1: Valve 1 is closed and Valve 2 is opened, exposing the gas to the sorbent. The sorbent isothermally reaches the equilibrium uptake.

Step 2: Valve 2 is closed, Valve 3 is opened. The temperature and pressure are changed along a specified path through this thermodynamic space. The desorbed gas leaves through the outlet check-valve, Valve 4.

Step 3: Valve 3 is closed, Valve 1 is opened, and the piston is drawn back. The system returns to the initial temperature and pressure.

To approximate atmospheric gas, we consider a binary mixture of N_2_ and CO_2_. It is important to clarify that, for the purpose of all the subsequent analyses in this work, the model will use absolute adsorption as the thermodynamically relevant measure. Absolute adsorption includes not only the molecules of gas interacting with the surface of the sorbent, but also the molecules of gas in the pores of the sorbent. For a more in-depth discussion of the measures of adsorption, see ref. 27.

#### 2.2.2 Energy balance

During Step 1, the (refreshed) sorbent is exposed to the new gas. For DAC this would be exposing it to the atmosphere, at ambient or slightly above ambient pressure. The sorbent adsorbs gas until it reaches equilibrium with the gas in the inlet source. Since adsorbing gas is exothermic, energy will be spent cooling the system to maintain the isothermal condition during adsorption. The gas being adsorbed changes the volume of the system. There is a work term associated with this gas contracting. Therefore the energy balance for Step 1 (assuming ideal gas behavior) is:1
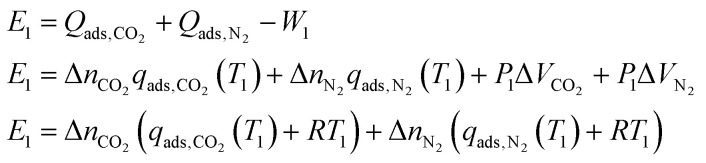
where *E*_1_ is the energy balance of Step 1, *Q*_ads_ is the heat of adsorption, *W*_1_ is the work of the gas contracting, *P*_1_ is the inlet pressure, Δ*V* is the change in volume of the gas, Δ*n* is the change in equilibrium uptake of the gas (*i.e.* the working capacity of that gas), *q*_ads_ is the molar heat of adsorption, *T*_1_ is the initial temperature, and *R* is the ideal gas constant. Note that we know the initial conditions (*T*_1_, *P*_1,CO_2__, *P*_1,N_2__) and can measure or predict *q*_ads_(*T*) (as seen in the previous section), but do not yet know the Δ*n* terms. We can get the initial equilibrium uptake (*n*_1,CO_2__ and *n*_1,N_2__) from the initial conditions, but the equilibrium uptakes at the end of the cycle (*n*_end,CO_2__, and *n*_end,N_2__) depend on the path taken during the desorption in Step 2. This is because, while the total pressure *P*_end_ can be chosen, the composition of the desorbing gas changes throughout the refresh cycle since (under our modeling assumptions) it maintains equilibrium with the sorbent and remaining sorbate. As we describe in more detail below, the composition of the captured gas at the end of the refresh cycle is determined by the integral over these small changes and is, therefore, highly path-dependent.

During Step 2, the system is now isolated from the atmosphere, and the temperature and pressure are changed, causing gas to desorb from the sorbent. This desorption is endothermic, requiring energy to be put into the system. There is a work term associated with the gas expanding. Energy is required to heat the gas in the system and the sorbent. Finally, there is the energy required to change the pressure. The total energy balance for Step 2 is therefore:2
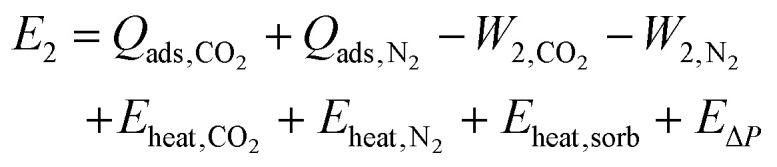


If we define a path *s* through (*T*, *P*)-space, then we can define the desorption or refresh cycle as:3
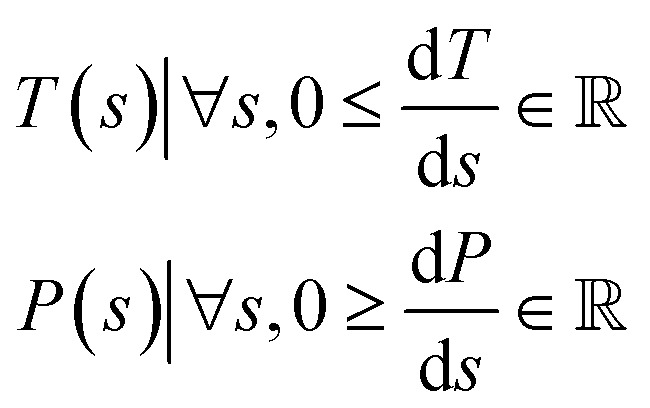
Meaning that *T* and *P* are both functions of the path *s*, under the constraint that for all *s* the change in *T* with respect to *s* is a non-negative real number and the change in *P* with respect to *s* is a non-positive real number. Then we can use eqn (3) to define any temperature-swing, pressure-swing (including vacuum swing), or combinations thereof refresh cycles for the desorption. Under this definition of the desorption path, eqn (2) becomes:4
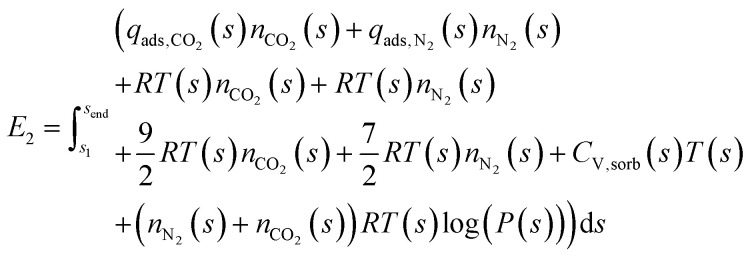
where *C*_V,sorb_(*s*) is the molar heat capacity at constant volume of the sorbent as a function of *s*. Note that here we assume that there is no change in volume of the sorbent with *T*, *P*, or as gas is adsorbed/desorbed. We approximate the heat capacity of the adsorbed CO_2_ and N_2_ with the ideal gas theory approximation for triatomic and diatomic gases, respectively.

During Step 3, the system returns from *T*_end_, and *P*_end_ to *T*_1_ and *P*_1_. Since the aim of this work is to develop performance metrics that depend on the intrinsic properties of materials in order to directly compare potential sorbent materials, we assume no energy recovery. Therefore the energy balance for Step 3 is:

5 *E*_3_ = 0

However, real DAC systems will be able to recover energy from the hot output gas cooling and the total pressure equilibrating. A simple example of this is using heat exchangers for the hot gas on the output of one gas separation column to warm another column. Since those terms are system design dependent, we will ignore them for this analysis.

The total energy balance for the refresh cycle is then simply the sum of the energies of each step:

6 *E*_Total_ = *E*_1_ + *E*_2_ + *E*_3_

We can then define our performance metrics for this intrinsic refresh cycle using the terms defined above. The purity of CO_2_ in the output is the mole fraction:7
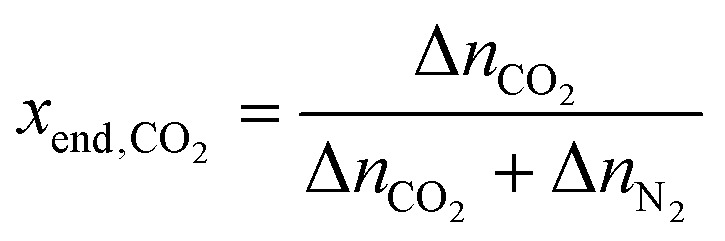


The intrinsic capture efficiency is how much CO_2_ was captured per unit energy:8
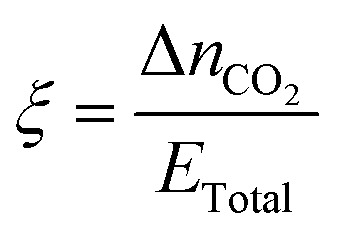


Alternatively, the inverse of *ξ* is the energy cost to capture an amount of CO_2_:9
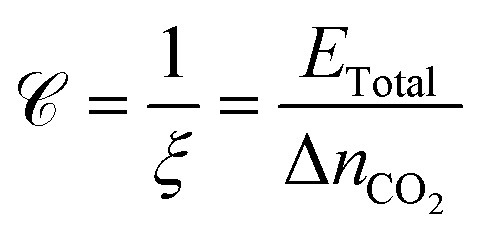
Note that in order to calculate *x*_end,CO_2__ and *ξ* we must first specify *s*, then determine *n*(*s*) and *q*_ads_(*s*) for each gas species, as well as *C*_V,sorb_(*s*), which were shown in the previous section.

## 3 Discussion

For ideal gases the enthalpy of mixing is zero – meaning that it takes zero work to separate ideal gases. This would mean that it would take zero work to capture the CO_2_ from the atmosphere. This would therefore imply that there is no upper limit on how much CO_2_ could be captured per energy. However, the Gibb's free energy of mixing for ideal gases is negative, which means that the mixed gases in atmosphere will not spontaneously separate. What we are modeling with the idealized piston described above is a way to keep track of all the thermodynamic terms for an adsorption based process. Indeed, if we recovered all the waste heat in Step 3 – if we replace eqn (5) to perfectly recover all the waste heat – then the total in eqn (6) would necessarily sum to zero. Real-world systems would likely employ some waste heat recovery and therefore have non-zero (but not perfectly efficient) *E*_3_ terms, and could therefore exceed our stated upper limit of capture efficiency. Since real-world waste heat recovery systems are not perfectly efficient and would depend on the process design rather than materials properties, for the goal of comparing materials based on their intrinsic properties we ignore any heat recovery. Therefore, this method provides an idealized upper bound on the purity and capture efficiency of sorbent based gas separations, in the absence of heat recovery – all of which is based off of intrinsic material properties.

The most critical information for this analysis is the equilibrium uptake of each component gas as a function of temperature and the other component gases. Unfortunately there is very little data on adsorption behavior in mixed gases.^15^ For the purposes of this initial work, we used thermodynamic extrapolation of *K*_H_ isotherms and IAST to obtain the equilibrium uptakes. These simplifying assumptions allowed us to screen through 11 660 MOF materials found in the CSD database. However, the analysis here could just as easily be applied to sorbents with strongly non-linear isotherms calculated from higher levels of theory or from experimental measurements, such as seen in amine-decorated MOFs.^28^ All that is needed is a way to describe the equilibrium uptake of each gas species as a function of temperature and each partial pressure. However, as mentioned earlier, there is a significant lack of experimental measurements of mixed gas adsorption,^15,16^ and a lack of non-linear adsorption models that account for temperature dependence (extrapolating in temperature from isotherms). This work further assumes the volume of the sorbent does not change with adsorption. Many MOF sorbents being considered for DAC applications are flexible and/or have nearly step-wise isotherms.^29^ To accurately consider flexible materials, the work associated with the sorbent volume change would need to be accounted for in the energy balance and specific heat calculations in eqn (1) and (4).

That being said, linear, non-interacting isotherms are good approximations for many MOF sorbents. For this study we considered the MOFs from the CSD.^13^ Out of the 11 600 materials studied in this work, there were 8 759 materials where the *K*_H_ are good approximations of the isotherms. This was determined by comparing the equilibrium uptake of N_2_ at 101.3 kPa (1 atm) and 300 K using the *K*_H,N_2__*versus* a direct GCMC calculation. A table categorizing all the completion mechanisms of our analysis is given in Section 3 of the SI.

**Fig. 2:**
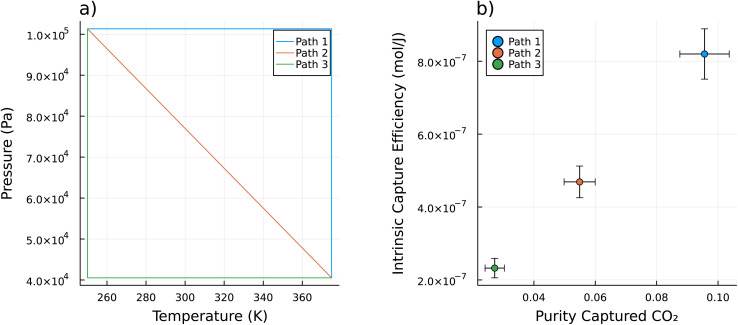
(a) A plot of 3 paths through (*T*, *P*)-space for the refresh cycle. These are three possible paths that could be used for Step 2 of the refresh cycle (as depicted in Fig. 1). Path 1 and 3 are the extreme examples. Path 1 can be thought of as heating first then pulling vacuum. Path 3 is the opposite, pulling vacuum first then heating. Path 2 heats and pulls vacuum concurrently, both at constant rates. (b) The performance metrics of the sorbent (LETQAE01_ion_b) along each of the 3 paths. For this material, given the binary mixture of CO_2_ and N_2_ at the inlet at 400 μmol mol^−1^ of CO_2_, both the intrinsic capture efficiency (from eqn (8)) and purity of the captured CO_2_ (from eqn (7)) are optimized by heating first then pulling vacuum. The error bars represent plus or minus one standard deviation as estimated from a Monte Carlo based uncertainty propagation from the initial GCMC calculations of both *K*_H,CO_2__, and *K*_H,N_2__ as well as the machine learning prediction of *C*_V_, up through the IAST calculations for mixed gas adsorption behavior, and the intrinsic DAC analysis from eqn (1) and (4) and ultimately to the performance metrics.

The performance of the sorbent depends on the path through thermodynamic space during the refresh cycle. Fig. 2a shows three example paths through (*T*, *P*)-space during Step 2. Each of these paths has the same starting and ending conditions, but take different paths through (*T*, *P*)-space to get there: heating first then pulling vacuum (Path 1, blue line), heating and pulling vacuum simultaneously (Path 2, orange line), and pulling vacuum first then heating (Path 3, green line). In Fig. 2b, we show the purity of the captured CO_2_ and the intrinsic capture efficiency for one sorbent material, LETQAE01_ion_b, for each of these paths. For this material, the performance in both metrics is optimized by heating first then pulling vacuum. The reason that the performance is path dependent is that, as the gas desorbs at one infinitesimal point along the path it is in equilibrium with the gas that desorbed at the previous infinitesimal point in the path.

**Fig. 3:**
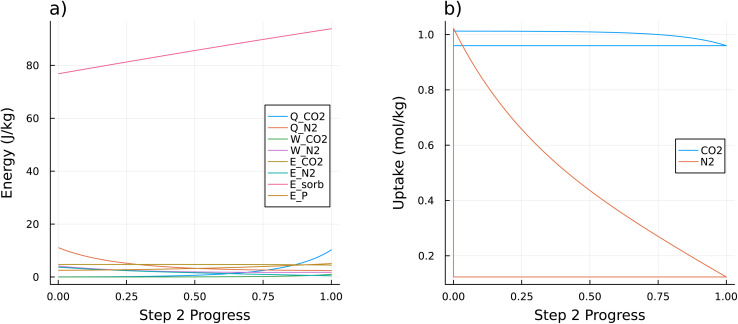
(a) A plot of each term in the energy balance from eqn (2) for the sorbent LETQAE01_ion_b along Step 2 of the refresh cycle specified by Path 2 from Fig. 2a. *Q*_CO__2__ and *Q*_N__2__ are the heats of adsorption of CO_2_ and N_2_, *W*_CO__2__ and *W*_N__2__ are the work associated with the change in volume of the desorbed CO_2_ and N_2_, *E*_CO__2__ and *E*_N__2__ are the energies required to heat the adsorbed CO_2_ and N_2_, *E*_sorb is the energy required to heat the sorbent, and *E*_*P* is the energy required to change the pressure. The total energy is clearly dominated by the *E*_sorb term for the energy required to heat the sorbent. (b) A plot showing the equilibrium uptakes (*n*_CO_2__(*s*) and *n*_N_2__(*s*)) for the sorbent LETQAE01_ion_b along the refresh cycle specified by path 2 from Fig. 2a. Since both *T* and *P* change concurrently at constant rates during Step 2 of this path, the *x*-axis is labeled as the progress along this path. The uptake along Step 1 is shown as the vertical line segment from low uptake to high uptake for each sorbate. The uptake along Step 2 is shown as the curve from the upper left to the bottom right for each sorbate. The uptake along Step 3 is shown as the horizontal line segment at constant uptake of each sorbate. This plot is a specific example of the generic plot shown in Fig. 1b.

Fig. 3a shows each of the terms in the energy balance from eqn (4) along the progress of Step 2 for Path 2 in Fig. 2a. The heat of adsorption of N_2_ decreases as the cycle progresses, while the heat of adsorption of CO_2_ increases as the cycle progresses. By far, the largest term in this energy balance is the energy required to heat the sorbent. Fig. 3b shows the equilibrium uptake of both CO_2_ and N_2_ along this refresh cycle, analogous to the diagram in Fig. 1b. The equilibrium uptake along Step 1 is the vertical portion, while the equilibrium uptake along Step 3 is the horizontal portion. The working capacities are differences between equilibrium uptakes at the start and end of Step 2 (equivalently, the length of the vertical portion for Step 1).

**Fig. 4:**
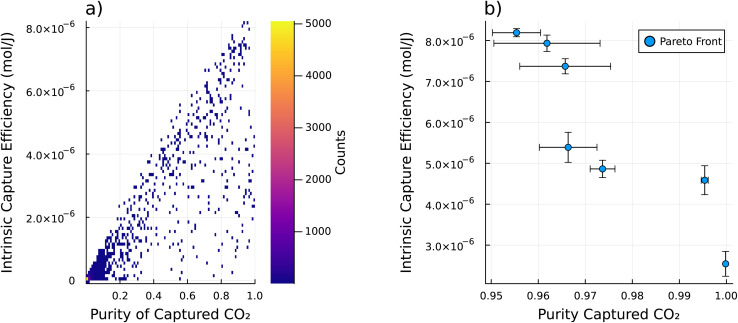
The intrinsic DAC analysis was performed on each sample in the database using Path 1 from Fig. 2a. A 2D histogram of the performance metrics for this analysis is shown in (a). Note that where there are no sorbents the histogram bin (pixel) is left transparent. The colorbar shows the count of how many sorbents occupy that bin of performance metrics. Note that there are many sorbents with poor performance, as shown by the few bright bins at the bottom left corner of the 2D histogram. This shows that there are many sorbents that do not perform well with this refresh cycle (as defined by Path 1 from Fig. 2a), while a few sorbents do perform well. (b) shows the Pareto front of part (a). This shows the set of Pareto optimal materials for this refresh cycle. Note that the error bars show plus or minus one standard deviation for each performance metric. This uncertainty was estimated from a Monte Carlo based uncertainty propagation from the uncertainties in the initial GCMC calculations of both *K*_H,CO_2__, and *K*_H,N_2__ as well as the machine learning prediction of *C*_V_, up through the IAST calculations for mixed gas adsorption behavior, and the intrinsic DAC analysis from eqn (1) and (4) and ultimately to the performance metrics.

We then consider a single path for the refresh cycle and examine the intrinsic refresh for all of the MOF materials in the CSD. This path is the same as Path 1 from Fig. 2a – which starts at 250 K and 101 325 Pa, warms isobarically to 350 K, then pulls vacuum isothermally to 40 530 Pa. The 2D histogram of the performance metrics of all of the sorbents considering this path is shown in Fig. 4a. While there is a high density of sorbents that perform poorly by both metrics (toward the bottom left), there are still some sorbents that have impressive performance (toward the top right). Fig. 4b shows the set of Pareto optimal materials for this refresh cycle, which are also detailed in Table 1. Given Path 1 from Fig. 2a and the approximated atmospheric conditions, these are the optimal sorbents to use with respect to the intrinsic capture efficiency *ξ*, and purity of the captured CO_2_*x*_end,CO_2__ based on their intrinsic materials properties.

**Table 1:** Table of the Pareto optimal materials given a single path (specifically Path 1 from Fig. 2a). With intrinsic capture efficiency *ξ* in (μmol J^−1^), purity of the captured CO_2_*x*_end_, and working capacity Δ*n* in (mol kg^−1^). Note that the number in the parenthesis represents the uncertainty in the previous two digits of the nominal value

Name	*ξ*	*x*_end_	Δ*n*_CO_2__	Δ*n*_N_2__
jacs.6b06759_ja6b06759_si_003_clean	8.191(99)	0.9554(51)	13.7(17)	0.632(10)
ja5b02999_si_002_clean	7.93(20)	0.962(11)	12.6(32)	0.4641(17)
RAVXIX_clean	7.37(18)	0.9657(97)	33.2(70)	1.115(16)
PUPXII01_clean	5.39(37)	0.9664(61)	1.58(26)	0.0534(13)
ZADDAJ_clean	4.86(21)	0.9737(26)	1.42(14)	0.03802(92)
VAXHOR_clean	4.59(35)	0.99539(74)	1.05(17)	0.00473(16)
BOMCUB_charged	2.54(30)	0.999839(29)	0.326(53)	0.0000511(19)

**Fig. 5:**
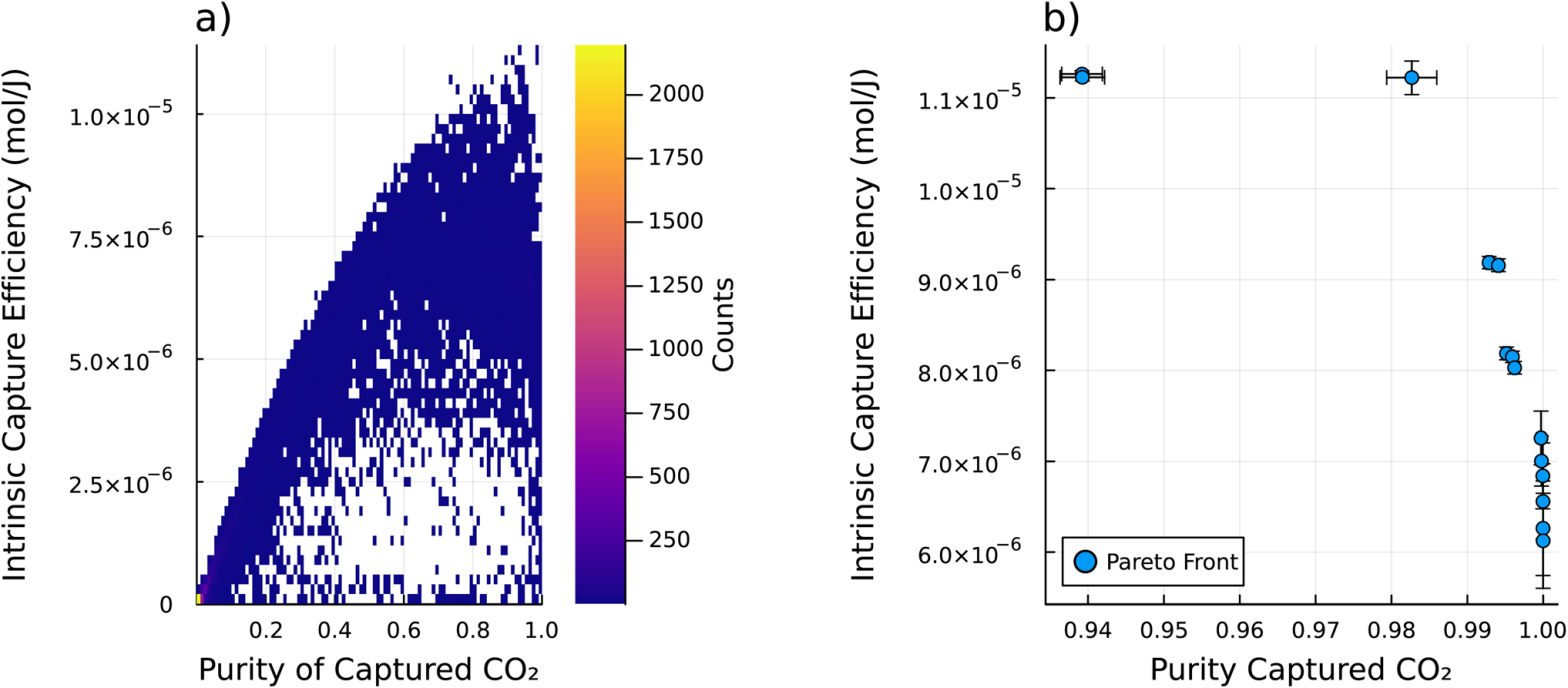
For each sorbent material in the database, we found Pareto optimal refresh paths within the bounds defined by eqn (10) based on the intrinsic DAC analysis. (a) shows A 2D histogram of the performance metrics for this analysis. Note that each sorbent material could have multiple Pareto optimal paths. The histogram shows the performance metrics for all Pareto optimal paths for all materials in the database. The colorbar shows the count of how many combinations of sorbent and refresh path occupy that bin of performance metrics, where there are none the bin (pixel) is left transparent. Despite individually optimizing the refresh paths, there are many sorbents with poor performance, as shown by the few bright bins in the bottom left of the histogram. (b) shows the Pareto front of part (a). This shows the set of Pareto optimal sorbents with their respective Pareto optimal refresh paths. Note that the error bars show plus or minus one standard deviation for each performance metric. This uncertainty was estimated from a Monte Carlo based uncertainty propagation from the uncertainties in the initial GCMC calculations of both *K*_H,CO_2__, and *K*_H,N_2__ as well as the machine learning prediction of *C*_V_, up through the IAST calculations for mixed gas adsorption behavior, and the intrinsic DAC analysis from eqn (1) and (4) and ultimately to the performance metrics.

Next, we consider optimizing the path for each material. The refresh path need only be monotonic in temperature and total pressure. However, to simplify the parametrization of the path for the purposes of this study, we narrow the search space to only consider linear paths between starting point (*T*_1_, *P*_1_) and ending point (*T*_end_, *P*_end_). To enforce the monotonic paths we use the priors:10
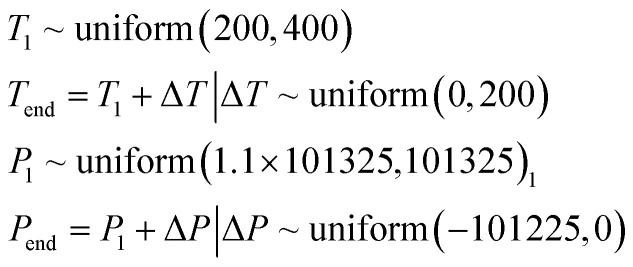
with *T* in Kelvin, and *P* in Pascal. We search for Pareto optimal refresh paths (Pareto optimal *T*_1_, Δ*T*, *P*_1_, and Δ*P*), for each material in the database. Each sorbent could have several Pareto optimal refresh paths – multiple paths that find the optimal tradeoff between *ξ* and *x*_end_ for each sorbent. Fig. 5a shows a 2D histogram of the performance metrics of all the Pareto optimal paths for all the MOFs in the database. The best performing materials are those in the top right of this figure. Despite the optimization there is high concentration of materials that do not perform well by either metric (as seen by the few bright histogram bins in the bottom left of Fig. 5a), which shows that these materials are likely poor candidates for DAC. However, in comparison to Fig. 4a, there is a much higher density of sorbents that perform well. This shows the utility of optimizing the refresh path for each sorbent. Fig. 5b shows the set of Pareto optimal sorbents with their respective Pareto optimal refresh paths, which are also summarized in Table 2. It is interesting to note that for many materials, refresh paths can be optimized to capture nearly pure CO_2_. Furthermore, the capture efficiencies achieved after the path optimization are much higher than those seen in the single path considered in Fig. 4. There are a few sorbents with intrinsic capture efficiencies above 1.0 × 10^−5^ mol J^−1^ for their Pareto optimal paths. To put some of the capture efficiency values in perspective, the average CO_2_ emissions by the US grid is about 2.46 × 10^−6^ mol J^−1^ according to the US Energy Information Administration^30^ – meaning at these idealized performances, many sorbents could potentially enable net-negative emissions with systems powered by the US grid. Of course these idealized efficiencies do not account for the real-world efficiencies of DAC systems – which would incur losses due to friction and other non-idealities, but could also include waste heat recovery. McQueen *et al.*^2^ states example systems currently operating with capture efficiencies of 2.27 × 10^−6^ mol J^−1^. However, that system was mostly powered by natural gas, which results in net positive emissions. In any case, this analysis provides an idealized upper limit on the capture efficiency which shows that real-world systems have potential for efficiency gains.

**Table 2:** Table of the Pareto optimal materials and their Pareto optimal paths. With intrinsic capture efficiency *ξ* in (μmol J^−1^), purity of the captured CO_2_*x*_end_, working capacity Δ*n* in (mol kg^−1^), temperature *T* in (K), and pressure *P* in (Pa). Note that the number in the parenthesis represents the uncertainty in the previous two digits of the nominal value

Name	*ξ*	*x*_end_	Δ*n*_CO_2__	Δ*n*_N_2__	*T*_1_	*T*_end_	*P*_1_	*P*_end_
VANNIK_clean	11.259(38)	0.9392(27)	29.1(11)	1.878(50)	200.7	384.3	101 950	60 838
VANNIK_clean	11.223(41)	0.9392(29)	30.0(13)	1.935(55)	200.9	371.2	106 080	29 443
LETQAE01_ion_b	11.22(18)	0.9827(33)	137(24)	2.346(30)	200.2	391.2	101 786	61 381
BEVQID_clean	9.186(66)	0.99287(37)	3.17(13)	0.02273(65)	210.5	308.7	100 868	10 036
BEVQID_clean	9.156(68)	0.99409(29)	3.75(16)	0.02224(67)	210.2	338.9	97 326	7838
FASJAL_clean	8.186(69)	0.99517(36)	4.20(29)	0.02032(58)	236.6	316.7	99 144	2432
FASJAL_clean	8.148(62)	0.99594(23)	4.64(25)	0.01890(51)	235.3	344.9	87 503	4609
FASJAL_clean	8.028(69)	0.99623(22)	4.78(24)	0.01804(44)	235.0	364.1	82 466	3243
IFUDAO_charged	7.26(29)	0.999715(46)	1.61(22)	0.000450(19)	217.7	307.3	100 360	9733
IFUDAO_charged	7.00(28)	0.999767(31)	2.02(24)	0.000463(18)	217.7	345.7	102 382	14 448
MAXHEA_clean	6.84(36)	0.9999511(83)	0.99(15)	0.0000471(26)	213.1	300.3	110 012	12 457
MAXHOK_clean	6.56(41)	0.9999731(52)	0.93(15)	0.0000243(12)	212.9	302.5	93 225	9613
MAXHOK_clean	6.26(52)	0.9999739(63)	1.04(20)	0.0000259(12)	213.8	331.7	101 126	17 833
PARFOF_clean_h	6.12(52)	0.9999911(19)	1.14(21)	0.00000974(45)	203.6	311.7	102 499	8995

**Fig. 6:**
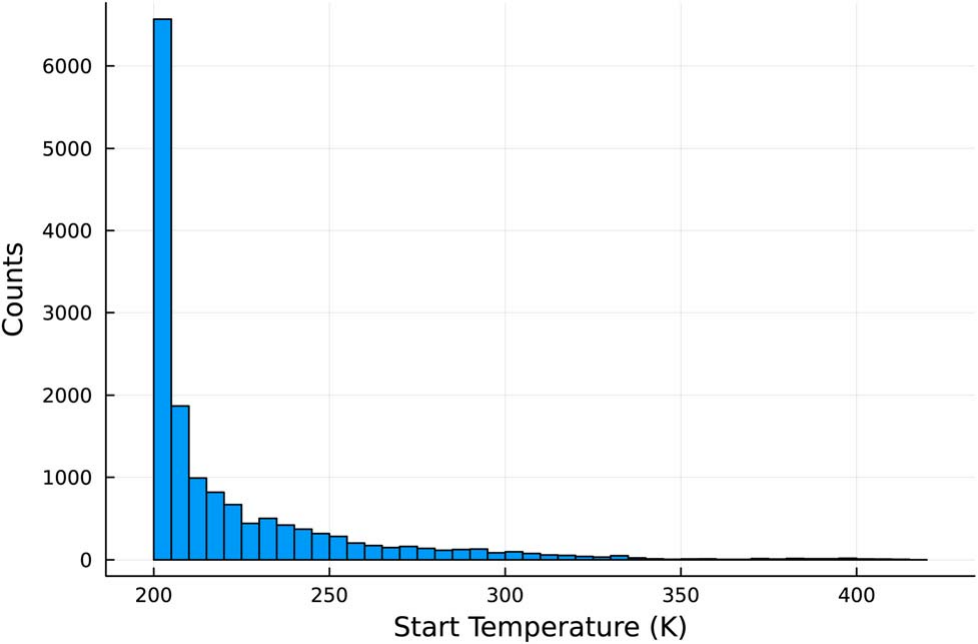
For each sorbent material in the database, we found Pareto optimal refresh paths within the bounds defined by eqn (10) based on the intrinsic DAC analysis. This shows histogram of the starting temperature (*T*_1_) of those refresh paths. Each sorbent could have multiple Pareto optimal paths. This histogram shows the distribution of *T*_1_ for all the Pareto optimal paths for all the sorbents in the database. Also note that 200 K was chosen as the lower limit for *T*_1_, since that is well outside likely practical operating conditions for DAC facilities. Most of the optimized refresh paths start well below ambient conditions.

**Fig. 7:**
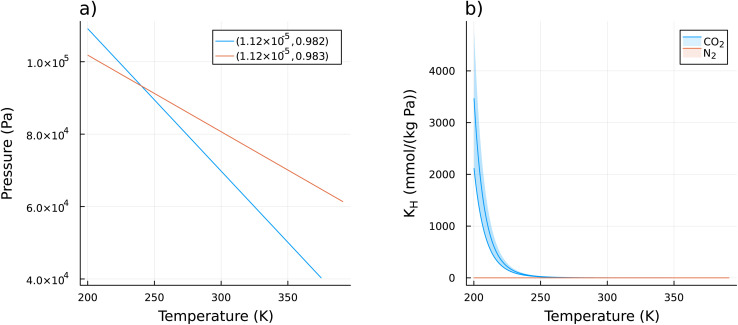
For the sorbent LETQAE01_ion_b we discovered Pareto optimal refresh paths. These paths are shown in (a) and labeled with the corresponding intrinsic DAC performance metrics: intrinsic capture efficiency *ξ* (mol J^−1^) and purity of captured CO_2_*x*_end_, respectively (*ξ*, *x*_end_). Part (b) shows the temperature dependence of the Henry's constant (*K*_H_) for CO_2_ and N_2_. As discussed in Section 2.1.1, the GCMC simulations were performed at 350 K for *K*_H,CO_2__, and 300 K for *K*_H,N_2__ then extrapolated in *β*-space to arbitrary temperatures. The shaded region shows two standard deviations from the mean, as estimated from a Monte Carlo based uncertainty propagation. Note how *K*_H,CO_2__ rapidly increases at lower temperatures, while *K*_H,N_2__ remains comparatively constant.

One of the main conclusions from this analysis is that it is greatly beneficial to start cold. Fig. 6 shows a histogram of all the starting temperatures (*T*_1_ from eqn (10)) of all the Pareto optimal paths for all the materials considered. Most of the materials' adsorption performance were optimized by lowering the start temperature below ambient. For this study we chose the lower limit of 200 K for the starting temperature to be somewhat outside the practical operating conditions of most DAC facilities, so as to not impose any undue restrictions. Yet the performance of many sorbents was often optimized at this hard limit, suggesting that the performance of some sorbents could be further optimized by even lower starting temperatures. We can gain some insight as to why the performance is optimized at low starting temperatures by inspecting the Pareto optimal paths of one material, LETQAE01_ion_b. Fig. 7a shows the Pareto optimal paths for this material, while Fig. 7b, shows the temperature dependence of the Henry's constants for the relevant temperature ranges. There is a dramatic increase in the *K*_H,CO_2__ as the temperature is lowered below 250 K, while the *K*_H,N_2__ remains comparatively constant. From inspection of eqn (7), the purity is optimized when *n*_N_2__(*s*) = constant or equivalently Δ*n*_N_2__ = 0. From inspection of eqn (1) and (4), if *n*_N_2__(*s*) = constant then the terms associated with the heat of adsorption (*Q*_ads,N_2__) of and work of adsorption (*W*_2,N_2__) of N_2_ tend toward zero, leaving only the small energy penalty associated with heating the adsorbed N_2_. This means that if *n*_N_2__(*s*) = constant then only CO_2_ is adsorbed or released, and some of the energy costs are eliminated. The implication of this is that selectivity for CO_2_ at any one point in the adsorption cycle is grossly incomplete information. Nor is the working capacity of CO_2_ sufficiently informative of the performance. This is evident in Table 2 where the working capacity of CO_2_ spans more than three orders of magnitude between the Pareto optimal materials despite fairly similar performance. The purity of the captured CO_2_ depends on the working capacity of CO_2_ and, crucially, the working capacity of N_2_. Since the dominant term in the energy balance is typically the heating of the sorbent material (as seen in Fig. 3), the capture efficiency is optimized by increasing the working capacity of CO_2_ and/or lowering the *C*_V,sorb_.

## 4 Conclusions

In this work we developed performance metrics for sorbents for DAC based on intrinsic materials properties. These metrics provide an idealized upper limit on the capture efficiency and the purity of the captured CO_2_. In order to evaluate these metrics the main information required is the equilibrium uptake of each of the gas species being considered as a function of the thermodynamic parameters (which include each independent partial pressure and temperature). In order to complete the energy balance, the sorbent materials properties that affect the relevant terms are needed. The most important of these – for refresh cycles involving temperature swings – is the sorbent heat capacity, which is typically the dominant contribution to the energy balance. In order to evaluate these metrics on the majority of the 11 660 MOF materials found in both the CSD and CoreMOF databases, we estimated the equilibrium uptake using IAST and GCMC calculated Henry's constants. We approximated atmospheric conditions with 400 μmol per mol CO_2_ with the balance of N_2_. We obtained estimates of the sorbent heat capacity at specific temperatures using pre-trained XGBoost models from ref. 23. We then interpolated and extrapolated these estimates to arbitrary temperatures using a heteroscedastic Gaussian Process regressor. These metrics can be used to find Pareto optimal sorbents for a given DAC refresh cycle – which could be useful in optimizing sorbent materials for existing DAC systems. Alternatively, these metrics could be used to find the Pareto optimal DAC refresh cycle for particular sorbents – which could be used to design DAC systems to take advantage of available sorbents. We used our simplified model to find the (linearly constrained) Pareto optimal refresh paths through (*T*, *P*)-space for the majority of the MOFs in the CSD database. With these results and an inspection of the energy balance it is clear that neither the selectivity at any one point along the cycle, nor the working capacity for CO_2_ are sufficient to describe the performance of the sorbent for DAC. We also show that due to the rapid change in the equilibrium uptake of CO_2_ at lower temperatures, it is beneficial to start the refresh cycles at lower temperatures. Many Pareto optimal refresh cycles start cold. We demonstrate the evaluation of these metrics for temperature–pressure swing refresh cycles, in a binary approximation of atmospheric conditions, and no waste heat recovery. However, it would be straightforward to include terms (in eqn (1) and (4)) for other refresh paths (*e.g.* electro-swing), for additional gas species (*e.g.* H_2_O, O_2_, as is shown in the SI), or for energy recovery systems in real-world facilities (by adjusting eqn (5)). Furthermore, while we used some simplified linear isotherms, many promising sorbents for DAC have highly non-linear (sometimes step-wise) adsorption curves.^28,29^ As we show in the derivation presented in the SI, these non-linear sorbents could be easily evaluated with the metrics we develop here if the mixed-gas temperature-dependent adsorption behavior is well described, but unfortunately that data is not currently available for most materials. This work, therefore, highlights the need for further studies on the equilibrium uptake as a function of temperature and in mixed gas environments.

## Disclaimer

Any mention of commercial products in this report is for information only; it does not imply recommendation or endorsement by NIST.

## Author contributions

AM initially conceptualized the study, developed the thermodynamic models and derived the metrics, developed the Julia code base for those metrics, ran GCMC and Intrinsic DAC cycle analysis calculations, and wrote the initial draft. DWS wrote the FEASST scripts for the GCMC calculations, provided feedback on the equilibrium uptake and thermodynamic models and derivations, and provided insight into the interpretation of the results. BD helped develop the Julia code base. KC and DLOM helped refine and guide the development of the study as part of the team led by DLOM. All authors contributed to the editing of the manuscript.

## Conflicts of interest

There are no conflicts to declare.

## Supplementary Material

SC-016-D5SC06099K-s001

SC-016-D5SC06099K-s002

## Data Availability

The code used to implement the intrinsic DAC cycle analysis is available as a Julia package at: https://github.com/usnistgov/IntrinsicDACCycle. The results of the GCMC calculations and intrinsic DAC cycle calculations used in this study are available at https://doi.org/10.5281/zenodo.16799659. Supplementary information: Derivation of the equilibrium uptake along the refresh path for linear isotherms and IAST, a correction for the real-world case of finite volume of the desorption chamber, the generic case for non-linear adsorption curves, details about the molecular simulations, and details summarizing the completion mechanisms of the refresh path calculations. See DOI: https://doi.org/10.1039/d5sc06099k.
