# Supplementary material for: Intrinsic direct air capture
Source: Chem Sci. 2025 Sep 10;16(39):18352–63. doi: 10.1039/d5sc06099k (PMC12421324; doi:10.1039/d5sc06099k)
Supplement: SC-016-D5SC06099K-s001 [file SC-016-D5SC06099K-s001.pdf]

Supplementary Information (SI) for Chemical Science.
This journal is © The Royal Society of Chemistry 2025

Intrinsic Direct Air Capture: Supplemental Information

Austin McDannald,[*,†,‡] Daniel W. Siderius,[¶,§] Brian DeCost,[†,‖] Kamal Choudhary,[⊥,#] and Diana L. Ortiz-Montalvo[†,@]

†*Materials Measurement Science Division, National Institute of Standards and Technology, Gaithersburg MD, USA*

‡*ORCID: 0000-0002-3767-926X*

¶*Chemical Sciences Division, National Institute of Standards and Technology, Gaithersburg MD, USA*

§*ORCID: 0000-0002-6260-7727*

‖*ORCID: 0000-0002-3459-5888*

⊥*Materials Science and Engineering Division, National Institute of Standards and Technology, Gaithersburg MD, USA*

#*ORCID: 0000-0001-9737-8074*

@*ORCID: 0000-0001-7293-4476*

E-mail: austin.mcdannald@nist.gov

1 Derivation Intrinsic DAC Cycle

In this section we show the derivation of how we obtain the equilibrium uptake at each point along the desorption path (Step 2 from Figure 1a in the main text). In Section 1.1 we show the derivation using Ideal Adsorbed Solution Theory. In Section 1.2 we show a correction for the real-world case of a desorption chamber with finite volume. Lastly, in Section 1.3 we

show the generalized derivation for generic functions for the equilibrium uptake and arbitrary number of species in the gas mixture.

1.1 Derivation using Ideal Adsorbed Solution Theory

Initially, after the adsorption step, the material is in equilibrium with the inlet conditions. Then the material is isolated from the inlet and the thermodynamic conditions (i.e., temperature T, and total pressure P) are changed to desorb the gasses. In our idealized system, there is no volume to the outlet (an idealized check-valve) so the composition of gas that desorbs is the composition of the outlet (we show later, in Section 1.2 how to correct for this). Therefore, the equilibrium uptake and composition of gas that is desorbed by changing the conditions are determined from equilibrium with the material using Ideal Adsorbed Solution Theory (IAST). In the limit of low pressure, the equilibrium uptake of a gas is linear with partial pressure, the slope of which is the Henry Constant. As discussed in the main text, the conditions of Direct Air Capture refresh cycles are typically sufficiently low pressure that Henry Constant isotherms is a good prediction. The Henry constants are a function of temperature, and therefore change over the refresh cycle. IAST can be solved analytically with these linear, Henry Constant isotherms.

To determine the equilibrium uptake during desorption (and consequently the moles of each gas species in the outlet) along the refresh path through thermodynamic parameter space, we can iteratively solve the IAST equations for small step changes in the conditions.
Given: the initial Henry Constants for CO_2 ($K_{H,1,CO_2}$), and N_2 ($K_{H,1,N_2}$), initial Pressure P_1, and initial CO_2 concentration x_{1,CO_2}.
Find: the equilibrium uptake of CO_2 and N_2 during adsorption. Then find the new CO_2 concentration x_{2,CO_2} during the desorption phase after isolating from the inlet and at the new conditions with $K_{H,2,CO_2}$, and $K_{H,2,N_2}$, and Pressure P_2.

The initial absolute equilibrium uptake of CO_2 is:

$$\begin{aligned} n_{1,CO_2} &= K_{H,1,CO_2} P_{CO_2} \\ &= K_{H,1,CO_2} P x_{1,CO_2} \end{aligned} \tag{1}$$

The initial absolute equilibrium uptake of N_2 is:

$$\begin{aligned} n_{1,N_2} &= K_{H,1,N_2} P_{N_2} \\ &= K_{H,1,N_2} P(1 - x_{1,CO_2}) \end{aligned} \tag{2}$$

Next, the system is isolated from the inlet and the thermodynamic conditions are changed desorbing some of the gas. The change in equilibrium uptake of each species is:

$$\begin{aligned} d_{CO_2} &= n_{2,CO_2} - n_{1,CO_2} \\ d_{N_2} &= n_{2,N_2} - n_{1,N_2} \end{aligned} \tag{3}$$

where n_{2,CO_2} and n_{2,N_2} are as yet unknown.

Since there is no volume of the outlet (idealized check valve), the change in equilibrium uptake (the gas that is desorbed: $d_{CO_2} + d_{N_2}$) is the gas of the outlet. The new concentration of CO_2 is therefore:

$$\begin{aligned} x_{2,CO_2} &= \frac{d_{CO_2}}{d_{CO_2} + d_{N_2}} \\ &= \frac{n_{2,CO_2} - n_{1,CO_2}}{n_{2,CO_2} - n_{1,CO_2} + n_{2,N_2} - n_{1,N_2}} \\ &= \frac{K_{H,2,CO_2} P_2 x_{2,CO_2} - n_{1,CO_2}}{K_{H,2,CO_2} P_2 x_{2,CO_2} - n_{1,CO_2} + K_{H,2,N_2} P_2 (1 - x_{2,CO_2}) - n_{1,N_2}} \end{aligned} \tag{4}$$

Rearranging Eq. 4, we get:

$$K_{H,2,CO_2}P_2x_{2,CO_2} - n_{1,CO_2} = x_{2,CO_2}(K_{H,2,CO_2}P_2x_{2,CO_2} - n_{1,CO_2} + K_{H,2,N_2}P_2(1 - x_{2,CO_2}) - n_{1,N_2}) \tag{5}$$

Or:

$$\begin{aligned} 0 =& (K_{H,2,CO_2}P_2 - K_{H,2,N_2}P_2)x_{2,CO_2}^2 \\ &+ (K_{H,2,N_2}P_2 - K_{H,2,CO_2}P_2 - n_{1,CO_2} - n_{1,N_2})x_{2,CO_2} \\ &+ n_{1,CO_2} \end{aligned} \tag{6}$$

This is the classic quadratic equation with:

$$\begin{aligned} A &= K_{H,2,CO_2}P_2 - K_{H,2,N_2}P_2 \\ B &= K_{H,2,N_2}P_2 - K_{H,2,CO_2}P_2 - n_{1,CO_2} - n_{1,N_2} \\ C &= n_{1,CO_2} \end{aligned} \tag{7}$$

Therefore the new CO_2 concentration is given by:

$$x_{2,CO_2} = \frac{-B \pm \sqrt{B^2 - 4AC}}{2A} \tag{8}$$

Of the 2 roots to Equation 8 we choose the solution in the physically meaningful range: $x_{2,CO_2} \in [0,1]$ AND closest to x_{1,CO_2} since each step is a small change in thermodynamic conditions. We can then determine the new equilibrium uptake for CO_2 and N_2 with:

$$\begin{aligned} n_{2,CO_2} &= K_{H,2,CO_2}P_2x_{2,CO_2} \\ n_{2,N_2} &= K_{H,2,N_2}P_2(1 - x_{2,CO_2}) \end{aligned} \tag{9}$$

The equilibrium uptake during the desorption process can then be calculated for arbitrary paths through thermodynamic parameter space by iteratively solving Eq.s 8 and 9 for small

steps. Note that this derivation assumes monotonic decreases in P as well as monotonic decreases in K_{H,CO_2} and K_{H,N_2} along the refresh path.

1.2 Derivation with Finite Volume

Note that for real systems the outlet would have a known volume, which could be accounted for in Eq. 4 by including the appropriate terms in the nominator and denominator for the amount of CO_2 in already in the outlet and total amount of gas already in the outlet, respectively. Here we introduce the new terms and show how to account for known finite volume of the outlet.

The initial absolute equilibrium uptake of CO_2 is:

$$\begin{aligned} n_{1,CO_2} &= K_{H,1,CO_2} P_{CO_2} \\ &= K_{H,1,CO_2} P x_{1,CO_2} \end{aligned} \tag{10}$$

The initial absolute equilibrium uptake of N_2 is:

$$\begin{aligned} n_{1,N_2} &= K_{H,1,N_2} P_{N_2} \\ &= K_{H,1,N_2} P (1 - x_{1,CO_2}) \end{aligned} \tag{11}$$

The initial moles of CO_2 in the free volume is:

$$\begin{aligned} m_{1,CO_2} &= \frac{P_{CO_2} V}{RT} \\ &= x_{1,CO_2} \frac{PV}{RT} \end{aligned} \tag{12}$$

The initial moles of N_2 in the free volume is:

$$\begin{aligned} m_{1,N_2} &= \frac{P_{N_2} V}{RT} \\ &= (1 - x_{1,CO_2}) \frac{PV}{RT} \end{aligned} \tag{13}$$

The change in the equilibrium uptakes are:

$$\begin{aligned} d_{CO_2} &= n_{2,CO_2} - n_{1,CO_2} \\ d_{N_2} &= n_{2,N_2} - n_{1,N_2} \end{aligned} \tag{14}$$

The new concentration considering the finite volume is:

$$\begin{aligned} x_{2,CO_2} &= \frac{m_{1,CO_2} + d_{CO_2}}{m_{1,CO_2} + m_{1,N_2} + d_{CO_2} + d_{N_2}} \\ &= \frac{m_{1,CO_2} + n_{2,CO_2} - n_{1,CO_2}}{m_{1,CO_2} + m_{1,N_2} n_{2,CO_2} - n_{1,CO_2} + n_{2,N_2} - n_{1,N_2}} \end{aligned} \tag{15}$$

Substituting and re-arranging we get:

$$\begin{aligned} 0 =& (K_{H,2,CO_2} P_2 - K_{H,2,N_2} P_2) x_{2,CO_2}^2 \\ &+ (\frac{P_1 V}{RT_1} + K_{H,2,N_2} P_2 - K_{H,2,CO_2} P_2 - n_{1,CO_2} - n_{1,N_2}) x_{2,CO_2} \\ &+ (n_{1,CO_2} - m_{1,CO_2}) \end{aligned} \tag{16}$$

Similarly to Eq. 6, this is the classic quadratic equation with:

$$\begin{aligned} A &= K_{H,2,CO_2} P_2 - K_{H,2,N_2} P_2 \\ B &= \frac{P_1 V}{RT_1} + K_{H,2,N_2} P_2 - K_{H,2,CO_2} P_2 - n_{1,CO_2} - n_{1,N_2} \\ C &= n_{1,CO_2} - m_{1,CO_2} \end{aligned} \tag{17}$$

With these definitions of A, B, and C, we can find the new CO_2 concentration as before in Eq. 8 with:

$$x_{2,CO_2} = \frac{-B \pm \sqrt{B^2 - 4AC}}{2A} \tag{18}$$

As before, of the 2 roots we choose the solution in the physically meaningful range: $x_{2,CO_2} \in [0, 1]$ AND closest to x_{1,CO_2} since each step is a small change thermodynamic conditions. We

can then determine the new equilibrium uptake for CO_2 and N_2 with:

$$
\begin{aligned}
n_{2,CO_2} &= K_{H,2,CO_2} P_2 x_{2,CO_2} \\
n_{2,N_2} &= K_{H,2,N_2} P_2 (1 - x_{2,CO_2})
\end{aligned}
\tag{19}
$$

1.3 Generic Intrinsic Refresh Cycle

In Sections 1.1 and 1.2 we used linear Henry's constants and IAST to obtain the equilibrium uptake in the binary mixture of CO_2 and N_2. One advantage of using this framework, is that the uptake can be solved for analytically - as we have shown. However, in general, the Henry's constants may not describe the single component adsorption behavior well, the adsorbed gas species may interact invalidating the base assumptions of IAST, or there may be more than 2 gas species to consider. In this section we show how the Intrinsic DAC cycle analysis could be extended to become a generic Intrinsic Refresh Cycle for these more complicated gas separation problems. To keep the discussion generic we will refer to a multi-component gas mixture with species: A, B, C, ...

The initial absolute equilibrium uptake of each species are given by:

$$
\begin{aligned}
n_{1,A} &= n_A(T_1, P_{1,A}, P_{1,B}, P_{1,C}, ...) \\
n_{1,B} &= n_B(T_1, P_{1,A}, P_{1,B}, P_{1,C}, ...) \\
n_{1,C} &= n_C(T_1, P_{1,A}, P_{1,B}, P_{1,C}, ...) \\
&...
\end{aligned}
\tag{20}
$$

That is, the equilibrium uptake of each species is a function of the temperature and each independent partial pressure. While we control the total pressure P, the partial pressures

are determined by equilibrium with the sorbent. Re-writing in terms of P this becomes:

$$\begin{aligned} n_{1,A} &= n_A(T_1, x_{1,A}P, x_{1,B}P, x_{1,C}P, ...) \\ n_{1,B} &= n_B(T_1, x_{1,A}P, x_{1,B}P, x_{1,C}P, ...) \\ n_{1,C} &= n_C(T_1, x_{1,A}P, x_{1,B}P, x_{1,C}P, ...) \\ &... \end{aligned} \tag{21}$$

where:

$$x_{n,A} + x_{n,B} + x_{n,C} + ... = 1 | \forall n \tag{22}$$

At the beginning of the intrinsic refresh cycle the system will be in equilibrium with the known composition of the in-coming gas mixture, so the gas compositions $(x_{1,A}, x_{1,B}, x_{1,C}, ...)$ will be known and the equilibrium uptake $(n_{1,A}, n_{1,B}, n_{1,C}, ...)$ can be calculated. As the desorption step of the intrinsic refresh cycle begins, at each point along the path through thermodynamic parameters the system will be in equilibrium with the gas that desorbs from the sorbent at the infinitesimally previous point along the path. This can be solved for numerically by considering the change in equilibrium uptake.

The change in the equilibrium uptakes are:

$$\begin{aligned} d_A &= n_{2,A} - n_{1,A} \\ d_B &= n_{2,B} - n_{1,B} \\ d_C &= n_{2,C} - n_{1,C} \\ &... \end{aligned} \tag{23}$$

If there is a known finite volume of the desorption chamber to consider, then the initial moles

of each gas species in that finite volume is given by:

$$\begin{aligned} m_{1,A} &= (x_{1,A})\frac{PV}{RT} \\ m_{1,B} &= (x_{1,B})\frac{PV}{RT} \\ m_{1,C} &= (x_{1,C})\frac{PV}{RT} \\ &... \end{aligned} \tag{24}$$

We can then determine the new concentrations with:

$$\begin{aligned} x_{2,A} &= \frac{m_{1,A} + d_A}{m_{1,A} + m_{1,B} + m_{1,C} + ... + d_A + d_B + d_C + ...} \\ x_{2,B} &= \frac{m_{1,B} + d_B}{m_{1,A} + m_{1,B} + m_{1,C} + ... + d_A + d_B + d_C + ...} \\ x_{2,C} &= \frac{m_{1,C} + d_C}{m_{1,A} + m_{1,B} + m_{1,C} + ... + d_A + d_B + d_C + ...} \\ &... \end{aligned} \tag{25}$$

This can then be re-arranged to give:

$$\begin{aligned} 0 &= x_{2,A} - \frac{m_{1,A} + d_A}{m_{1,A} + m_{1,B} + m_{1,C} + ... + d_A + d_B + d_C + ...} \\ 0 &= x_{2,B} - \frac{m_{1,B} + d_B}{m_{1,A} + m_{1,B} + m_{1,C} + ... + d_A + d_B + d_C + ...} \\ 0 &= x_{2,C} - \frac{m_{1,C} + d_C}{m_{1,A} + m_{1,B} + m_{1,C} + ... + d_A + d_B + d_C + ...} \\ &... \end{aligned} \tag{26}$$

Equation 26 can then be solved with a root finding operation to find the new concentrations under the constraint of Equation 22 that the concentrations must sum to 1. This processes can then be followed iteratively at finite steps along the refresh path to obtain the equilibrium uptake for the intrinsic refresh cycle.

2 Molecular Simulations

The following section describes the molecular simulations that were used to generate pure-species adsorption isotherms and the isosteric heat of adsorption as well as consistency checks that yielded bounds on operating limits of the DAC cycle. We also point the reader to example simulations scripts in the associated SI content (file: `example_scripts.tgz` [update at time of proofs]) and provide essential instructions that may be used to reproduce our results.

2.1 Molecular Models

For the simulations that support present work, we used classical forcefields for both the adsorbate fluids (N_2 and CO_2) and the adsorbent materials (MOFs). N_2 and CO_2 were modeled by the respective TraPPE forcefield,[1] in which the model molecules have two (N_2) or three (CO_2) Lennard-Jones (LJ) sites and three point charges. In our work, the MOFs were modeled using all-atom forcefields, where each atom has an LJ site and a point charge. The structure of the MOFs were taken directly from the CoREMOF-2019 database.[2] LJ site parameters were assigned from the DREIDING[3] library and point charges were assigned using the mCBAC algorithm.[4] Cross-parameters for LJ sites were assigned using Lorentz-Berthelot combining rules. Electrostatic interactions were modeled using the Ewald summation method, with the Ewald damping parameter and number of Fourier vectors set by the recipe from DL_POLY[5] with a relative tolerance of 10^{-5}. Lastly, all LJ potentials and real-space terms in the Ewald summation were cut at 12 Å, with no tail correction.

2.2 Pure-species Adsorption Properties

As described in the main text, the pure-species adsorption isotherms of CO_2 and N_2 are represented by linear adsorption isotherms (cf. equations 1 and 2):

$$n_i\left(T, P_i\right) = K_{H,i}\left(T\right) P_i, \tag{27}$$

that is, the adsorption of adsorbate species i (n_i) as a function of temperature (T) at partial pressure of species i (Pi) is proportional to P_i, where the constant of proportionality is the Henry's Law Constant $K_{H,i}\left(T\right)$. $K_{H,i}$ may be computed for a model system using the Widom-insertion technique.[6,7] This technique is essentially a Monte Carlo Integration method to compute the average Boltzmann Factor of a single adsorbate molecule. The adsorbate is inserted into the adsorbent material at a random position with random orientation, the Boltzmann Factor is computed, and the sequence is repeated. In its base form, the Widom-insertion method yields the Henry's Law Constant at a single temperature. To allow for estimation of the adsorption isotherm at other temperatures, we employ a temperature extrapolation also based on the Widom-insertion technique[8] where, in addition to performing a Monte Carlo integration of the Boltzmann Factor, we also compute moments of the internal energy of the lone adsorbate that yield the extrapolation coefficients of a Taylor series. Then, $K_{H,i}$ can be computed via

$$K_{H,i}\left(\beta\right) \approx K_{H,i}\left(\beta_0\right) + \sum_{j=1}^{M} K_{i,j}\left(\beta_0\right)\left(\beta - \beta_0\right)^j \tag{28}$$

where we have switched to inverse temperature $\beta = 1/k_B T$ and $Ki, j\left(\beta_0\right)$ is the extrapolation coefficient of species i for moment j, computed at the original β_0. The extrapolation utilizes M coefficients, typically 10 to 20. The reader may consult Ref.[8] for full derivation of the temperature extrapolation equation and how the extrapolation coefficients are related to the Boltzmann Factor and energy moments.

A secondary output of the temperature extrapolation equation is the isosteric heat of adsorption, (q^{∞}_{ads}), which is, for our purposes, the heat released upon adsorption of one mole of adsorbate by the adsorbent material during the DAC cycle. This heat of adsorption is

$$q^{\infty}_{ads,i}\left(\beta\right) \;=\; \frac{1}{\beta} + \frac{\sum_{j=1}^{M} j \cdot K_{i,j}\left(\beta_0\right)\left(\beta - \beta_0\right)^{j-1}}{\sum_{j=0}^{M} K_{i,j}\left(\beta_0\right)\left(\beta - \beta_0\right)^{j}} \tag{29}$$

The Widom-insertion technique for computing $K_{H,i}$ is implemented in FEASST,[9] via Python scripts that are provided in the present SI content. The script is compatible with all FEASST versions 0.25.1 and lower; the specific Python script requires that FEASST be compiled with SWIG, which is not supported for later versions. To be precise, we ran the calculations with FEASST version 0.19.0, but more recent versions yield identical results. Lastly, the FEASST implementation of this calculation also estimates the uncertainty in $K_{H,i}$ and the $K_{i,j}$ extrapolation coefficients.

In the Python script, $K_{H,i}$ and $K_{i,j}$ for $M = 20$ are computed using a minimum of 10^6 trial insertions and a maximum of 5×10^7 trials, with an autoconvergence scheme that attempts to stop the simulation when specific criteria are met. For our calculation, the calculation stopped early when the relative standard uncertainty in $K_{H,i}$ is below 1 % and the relative standard uncertainty in $K_{i,10}$ is below 5 %.

Python scripts and all necessary support software apart from FEASST itself are in `Kh` subdirectory of the SI content archived in `example_scripts.tgz`. The individual files `n2_calc_kh.py` and `co2_calc_kh.py` are the example scripts for computation of $K_{H,N2}$ (300 K) and $K_{H,CO2}$ (350 K), respectively, for the adsorbent material `ZOYKAB_clean`. After compiling FEASST (using the SWIG option) and ensuring that the resultant `feasst` Python package is in the user environment, the script may be executed as

```
> python n2_calc_kh.py
```

and similar for `co2_calc_kh.py`. The scripts store results in JSON files that are indicated in the screen output.

2.3 N_2 adsorption at 300 K and 1 atm

To confirm that the Henry's Law Isotherm for N_2 in a particular MOF is adequate, we performed a simulation of N_2 in each MOF at 300 K and atmospheric pressure (1.01325 bar) to compute the adsorption uptake at those conditions. Then, if the Henry's Law isotherm of N_2 for the same material deviates by more than 10 %, the material is discarded and excluded from the DAC cycle analysis.

The simulation is a conventional Grand-canonical Monte Carlo (GCMC) simulation of adsorption using the potential models described earlier, run for 10^6 equilibration trials and 5×10^6 production trials. Our simulation used translation, rotation, and transfer (insertion and deletion, with equal probability) trial moves, with probability ratio 1:1:4. The only output of concern is the average number of nitrogen molecules. The box size was set by the MOF itself, the temperature was set to 300 K, and the N_2 chemical potential set to yield an external pressure of 1.01325 bar, assuming an ideal gas equation of state.

Python scripts are in the `N2_Atmospheric` subdirectory of the SI content in `example_scripts.tgz`. As discussed earlier, these scripts use FEASST with SWIG-library support. Our simulations were run using FEASST version 0.19.0. The example script, for the `ZOYKAB_clean` MOF may be executed as:

```
> python n2_atmosphere.py
```

The results are stored in a JSON file noted in the screen output. We note that the results in the data repository (`https://doi.org/10.5281/zenodo.14452152`) are reproduced precisely using this script and FEASST version 0.19.0. Due to evolution of the FEASST code base (e.g., subtle details of how the trials moves are selected and generated), later versions of FEASST will produce statistically equivalent results but will not match the output from version 0.19.0 precisely.

2.4 CO_2 Adsorption Capacity

To ensure that the estimates of CO_2 adsorption from Henry's Law are physically reasonable, we also performed simulations of CO_2 in each MOF at 200 K to estimate the maximum adsorption; i.e., the adsorption capacity of each MOF. When we optimize the DAC cycle, the CO_2 uptake at each statepoint is compared to this adsorption capacity and the cycle is not allowed to include statepoints that exceed the maximum CO_2 capacity. Essentially, we run a sequence of very short GCMC simulations at 200 K with increasing fugacity, compute the average number of CO_2 molecules in the MOF, and terminate the sequence when the uptake stops increasing. Additionally, each step in the sequence begins with the final configuration of the previous step, except for the first step that necessarily begins with an empty MOF. Thus, we approximate the maximum uptake without determining the precise pressure-uptake relationship.

Each individual simulation is a plain GCMC simulation at fixed temperature (here, 200 K) and fixed chemical potential (input via fugacity, serving as a proxy for pressure). The simulation begins empty for the first step and is seeded with the final configuration of the preceding simulation for all other steps. The GCMC simulation uses translation, rotation, and transfer (insertion and deletion with equal probability) trial moves with probability ratio 3:2:5; note that 50 % of trial moves are molecule transfers to encourage the MOF cell to fill to equilibrium. Each simulation steps uses 5000 equilibration and 5000 production trials; we acknowledge that this is a *very* short simulation. As noted, the objective is not the exact pressure-uptake relationship (the adsorption isotherm) but we are instead interested in the maximum capacity, regardless of the pressure at which the MOF saturates. Following the production trials, we compute the average number of CO_2 molecules and compare that average to the same ensemble average for the previous step; the sequence is terminated when that change is below 1 %.

Python scripts are in the `CO2_Capacity` subfolder of the SI content in `example_scripts.tgz`. As before, these scripts require FEASST with SWIG-library support. Unlike the N_2 simu-

lations, our CO_2 capacity simulations were run using FEASST version 0.22.0; the script is not compatible with version 0.19.0 The example script, for the `ZOYKAB_clean` MOF may be executed as:

```
> python co2_capacity.py
```

Results are stored in a JSON file noted in the screen output. If the script is run with version 0.19.0, the results will differ numerically but are statistically equivalent to those from later versions.

3 Refresh Path Calculation Completion Mechanism

For each of the 11 660 MOF materials in the CSD database, we applied our Intrinsic DAC cycle analysis using the models described in the main text, and search for Pareto optimal refresh paths. As described in the main text, we constrain these paths to be linear and monotonic between two points in (T, P)-space. We performed GCMC calculations to determine the Henry's constants (K_H), then use thermodynamic extrapolation of those to get the temperature dependence. We truncate the paths to regions with monotonically decreasing K_H with increasing temperature. Since the extrapolated Henery's constants of CO_2 at low temperatures can be very high, we further truncate the paths to where the equilibrium uptake of CO_2 from the Henry's constant isotherm is less than the saturation uptake of CO_2: $K_{H,CO_2} \times P_{CO_2} < n_{CO_2,sat.}$. We also eliminate non-adsorbing materials, *i.e.* where the CO_2 saturation uptake is zero. Since the concentration of CO_2 in the approximation of atmospheric conditions is low, and the majority of the total pressure is composed of N_2, we check that the linear Henry's constant isotherm for N_2 is a good approximation of the equilibrium uptake of N_2. We do this by comparing the equilibrium uptake at one atmosphere of N_2 as predicted by the Henry's constant to a direct GCMC calculation of the N_2 uptake. If $n_{N_2} \pm 2 \times \sigma_{n,N_2}$ from the direct GCMC calculation is within 10 % of $K_{H,N_2} \times P$, then we consider the Henry's constant a good approximation. Lastly, we eliminate materials where the Intrinsic DAC cycle optimizer failed. This can happen during the uncertainty propagation for the equilibrium uptake. We sample whole trends of $K_H(T)$ from the thermodynamic extrapolation in order to preserve the smoothness and monotonicity of the trend. Despite this, the sampled trend of $K_H(T)$ can lead to imaginary or complex valued compositions (solutions to Equation 8) due to numerical issues related to the step size in T affecting the step in K_H.

Table 1: Table of the completion mechanisms for the Pareto optimization of the refresh paths for the MOFs in the CSD database and the likely cause.

Completion Mechanism	Count	Likely Cause
Materials that failed K_H extrapolation	4	Insufficient statistics after GCMC trials
Materials with paths truncated to nothing	2	$\{$monotonic $K_H(T)$ $\}$ $\cap$ $\{ K_{H,CO_2} \times P_{CO_2} < n_{CO_2,sat.} \}$ eliminated all path steps
Non-adsorbing Materials	5	Saturation calc. did not adsorb, likely non-porous
Materials that failed close-enough test	2880	Non-linear $n_{N_2}(P)$ at 101325 Pa N_2
Materials that failed at optimizer	10	Samples of $K_H(T)$ change too fast for step size leading to imaginary roots of analytical IAST
Successfully optimized materials	8759	
Total	11660	

References

(1) Potoff, J. A.; Siepmann, J. I. Vapor-liquid equilibria of mixtures containing alkanes, carbon dioxide, and nitrogen. *AIChE J.* **2001**, *47*, 1676–1682.

(2) Chung, Y. G.; Haldoupis, E.; Bucior, B. J.; Haranczyk, M.; Lee, S.; Zhang, H.; Vogiatzis, K. D.; Milisavljevic, M.; Ling, S.; Camp, J. S.; Slater, B.; Siepmann, J. I.; Sholl, D. S.; Snurr, R. Q. Advances, Updates, and Analytics for the Computation-Ready, Experimental Metal–Organic Framework Database: CoRE MOF 2019. *J Chem Eng Data* **2019**, *64*, 5985–5998.

(3) Mayo, S. L.; Olafson, B.; Goddard, W. A. DREIDING: a generic force field for molecular simulations. *J Phys Chem* **1990**, *94*, 8897–8909.

(4) Zou, C.; Penley, D. R.; Cho, E. H.; Lin, L.-C. Efficient and Accurate Charge Assignments

via a Multilayer Connectivity-Based Atom Contribution (m-CBAC) Approach. *J Phys Chem C* **2020**, *124*, 11428–11437.

(5) Todorov, E. I.; Smith, W. "The DL_POLY User Manual" version 4.03. http://www.ccp5.ac.uk/DL_POLY/, Accessed 15 April 2022.

(6) Widom, B. Some Topics in the Theory of Fluids. *The Journal of Chemical Physics* **1963**, *39*, 2808–2812.

(7) June, R. L.; Bell, A. T.; Theodorou, D. N. Prediction of Low Occupancy Sorption of Alkanes in Silicalite. *J Phys Chem* **1990**, *94*, 1508–1516.

(8) Siderius, D. W.; Hatch, H. W.; Shen, V. K. Temperature Extrapolation of Henry's Law Constants and the Isosteric Heat of Adsorption. *Journal of Physical Chemistry B* **2022**, *126*, 7999–8009.

(9) Hatch, H. W.; Siderius, D. W.; Shen, V. K. Monte Carlo molecular simulations with FEASST version 0.25.1. *Journal of Chemical Physics* **2024**, *161*, 0–19.
